# Culture-Negative Emphysematous Pyelonephritis as a Complication of Uncontrolled Diabetes Mellitus: A Case Report

**DOI:** 10.7759/cureus.27856

**Published:** 2022-08-10

**Authors:** Deborah Omoleye, Muhammad A Israr, Faria Tazin, Salma Habib, Shreeya Desai, Camille Celeste Go, Ayobami Aranmolate, Odalys Frontela

**Affiliations:** 1 Research, Grandville Medical Center, Lagos, NGA; 2 Research and Academic Affairs, Larkin Community Hospital Palm Springs Campus, Hialeah, USA; 3 Family Medicine, East Liverpool City Hospital, East Liverpool, USA; 4 Internal Medicine, Institute of Applied Health Sciences (IAHS), Chittagong, BGD; 5 Family Medicine, Larkin Community Hospital Palm Springs Campus, Hialeah, USA; 6 Internal Medicine, Larkin Community Hospital Palm Springs Campus, Hialeah, USA

**Keywords:** antibiotic therapy, acute kidney injury, hospitalized patients, infectious disease pathology, general nephrology, outpatient family medicine, general internal medicine, e.coli, uncontrolled diabetes mellitus, complicated pyelonephritis

## Abstract

Emphysematous pyelonephritis (EPN) is a severe, necrotizing infection of the renal parenchyma. It is commonly found as a complication of uncontrolled diabetes mellitus (DM). EPN has a terrible prognosis unless promptly identified and treated. In this case study, a 38-year-old man with type 2 diabetes mellitus (T2DM) was admitted due to complaints of excruciating abdominal pain, vomiting, and non-adherent to his insulin medication. The patient was subsequently diagnosed with EPN. For most patients, the current course of treatment includes nephrectomy along with antimicrobial medications. In this case report, however, the patient improved with conservative treatment such as IV fluids, antibiotics, and blood glucose control.

## Introduction

Emphysematous pyelonephritis (EPN) is an acute bacterial infection in the parenchymal region of the kidneys [1,2]. The most common pathogens involved are *Escherichia coli *(*E. coli*),* Klebsiella pneumoniae*,* Proteus mirabilis*, *Clostridium septicum*,* Candida albicans*,* and *Pseudomonas [3]. In a favorable environment, these organisms cause fermentation of glucose and lactate to carbon dioxide which develops a necrotizing infection in the perinephric fat of the kidney [1,2]. Almost 90% of cases are found as a complication of uncontrolled diabetes mellitus (DM) or due to medication non-adherence [1,2]. Since patients with uncontrolled diabetes tend to have elevated blood sugar and glucosuria, these organisms, particularly *E. coli* thrive greatly in this environment. This is a case of a 38-year-old man with a 10-year history of poorly controlled type 2 diabetes mellitus (T2DM) and hypertension who presented with severe upper abdominal pain, vomiting, and signs of shock. The Computed tomography (CT) scans confirmed the diagnosis of EPN.

## Case presentation

A 38-year-old male came to the emergency department due to complaints of severe upper abdominal pain for the past 12 hours associated with five episodes of non-bilious, non-projectile vomiting. The patient denied any history of fever, changes in consciousness, or urinary or gastrointestinal symptoms. The patient has a history of T2DM and hypertension, with poor medication adherence for the past 10 years. The patient was non-compliant with his insulin regimen and has opted to take an African herbal supplement made of bitter kola there to four times a day in the past six years as an alternative to his insulin therapy. The patient was self-medicated with oral ciprofloxacin.

On physical examination, the patient was afebrile, tachycardic at 101 beats per minute, blood pressure of 152/110 mmHg, respiratory rate of 24 cycles per minute, and oxygen saturation of 98% on room air. Laboratory examinations were remarkable for an elevated random blood sugar of 312 mg/dL, hemoglobin A1c (HbA1c) 11.3%, and elevated neutrophil count of 85.6%. The rest of the laboratory findings including urine and blood cultures were unremarkable. The patient was treated with intravenous (IV) fluid, insulin, and antibiotics ceftriaxone and metronidazole.

**Figure 1:**
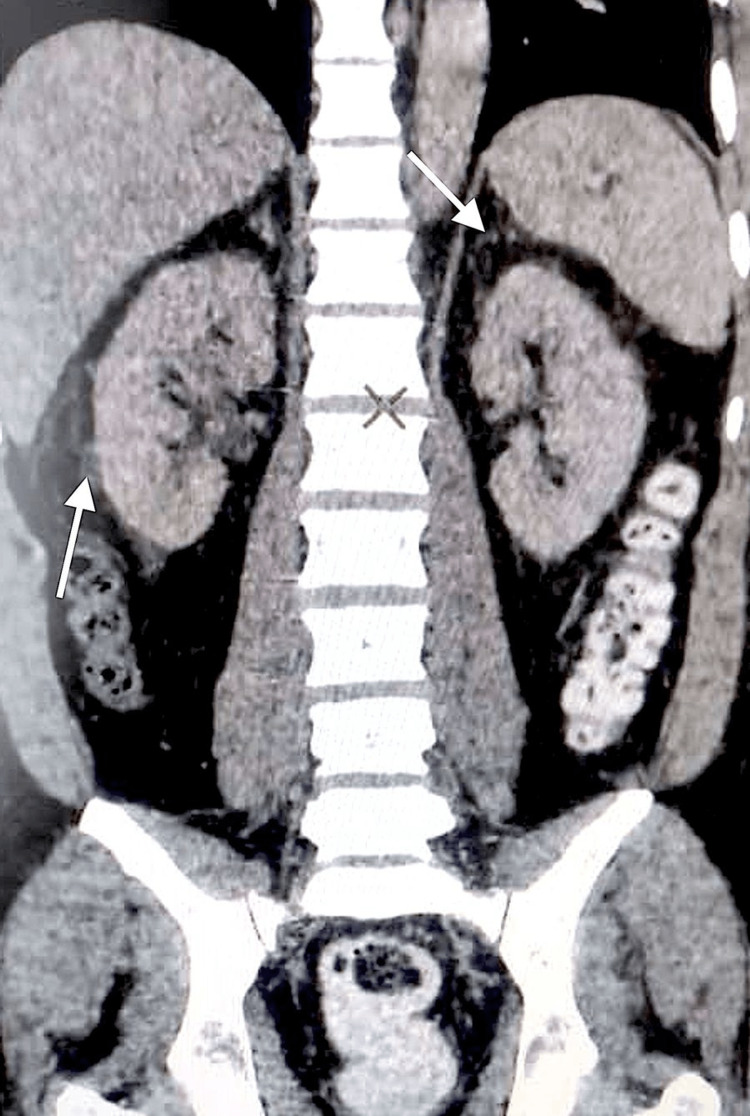
CT scan shows mild bilateral perinephric fat stranding (arrows).

**Figure 2:**
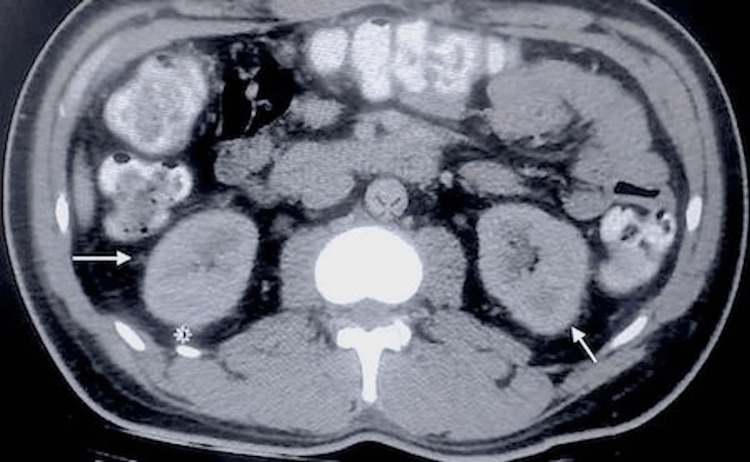
CT scan shows mild bilateral perinephric fat stranding (arrows).

On day two, the patient had hypotension and tachycardia, which were consistent with shock. He responded to IV fluids, however, his condition worsened on day three wherein he had four episodes of non-bilious, non-projectile vomiting associated with body weakness. Abdominal tenderness was noted in the epigastric area and rebound tenderness in the periumbilical region. IV levofloxacin was empirically added. As shown in Figures 1, 2, the CT scan confirmed mild perinephric fat strandings on bilateral kidneys, suggestive of EPN.

Urinalysis showed positive glucose and ketones. Treatment with intravenous levofloxacin, ceftriaxone, and metronidazole was continued for one week. The patient’s condition continued to improve and was subsequently discharged by day seven on a two-week course of oral cefixime, metronidazole and levofloxacin, insulin, and a blood pressure regimen.

## Discussion

EPN is a severe necrotizing infection that causes the accumulation of gas in the renal parenchyma, collecting system, and perirenal tissue and has high morbidity and mortality if left untreated [3]. It is a serious complication of DM [4]. Hyperglycemia with glucosuria, gas-forming bacteria, impaired tissue perfusion, and impaired neutrophil activity (reduced oxidative burst) in diabetics are responsible for the development of EPN [5]. It is essential to diagnose EPN early, especially in diabetic patients and the diagnosis is primarily made by non-contrast CT [6]. Gram-negative bacteria such as *E. Coli* are the most common pathogen, responsible for this infection. The underlying factors include high tissue glucose levels, which compromise tissue perfusion, and impaired neutrophil activity found in diabetic patients [2,7]. According to a meta-analysis, only 5-10.2% of EPNs are bilateral, with 52% of cases affecting the left kidney and 37.7% in the right [8]. With a female-to-male ratio of 4-6:1 and an average age of 57 years, EPN is prevalent in patients with T2DM and patients in immunocompromised states. These immunocompromised states include renal allograft, chronic steroid use, alcoholism, chronic kidney disease, or patients on chronic immunosuppressant medication [3,9]. EPN can be complicated by several diseases including emphysematous cystitis (gas in the wall of the urinary bladder) [10], emphysematous pyelitis (gas in the collecting system) [11], and shock [3]. According to the research, patients experiencing secondary shock following EPN have a mortality rate of 54% [3]. Patients with hemodynamic shock presenting with altered mentation, acute kidney injury requiring dialysis, and nutritional deficiency have a very high mortality rate. Polymicrobial infections and delay in empiric antibiotics are associated with higher mortality [12]. Additionally, in individuals with EPN, diabetic ketoacidosis is a significant predictor of death and people with thrombocytopenia can also experience poor outcomes [11,13]. According to Punatar et al., patients having a neutrophil-lymphocyte ratio greater than five at admission are at an increased risk of adverse outcomes by 1.8% [14]. The death rate is highest and extensive therapy is required when two or more factors are present [15].

Management includes broad-spectrum antibiotics, fluid resuscitation, glycemic control, bladder drainage with the catheter, and treatment of comorbid conditions. For severe EPN, invasive procedures such as percutaneous drainage, debridement of the infected area, and simple, complex, or radical nephrectomy may be warranted [16]. However, our patient was stabilized with the administration of IV fluids, antibiotics, glycemic control, and antihypertensives, which obviated the need for surgical intervention. Regular follow-up visits are necessary on discharge to ensure medication adherence and clinical resolution of EPN.

## Conclusions

EPN is a rare necrotizing infection of the renal parenchyma with a high mortality rate if not treated promptly and aggressively. Uncontrolled diabetic patients with poor antibiotic response are at higher risk of EPN therefore vigorous investigations including labs, imaging, and consultation should be performed to come up with a definitive diagnosis to treat the patients appropriately and on time. *E. coli* and other gas-forming bacteria are responsible for developing EPN in uncontrolled diabetic patients as these organisms thrive redundantly in a high glucose environment. In this case, the patient was non-compliant with his insulin regimen. Instead, the patient unsuccessfully self-treated himself with African herbal supplements to control his high blood sugar. Eventually, the patient developed EPN due to uncontrolled DM. Our patient improved significantly with aggressive inpatient medical management. However, surgical intervention such as nephrectomy may be needed in severe cases. It is necessary to educate every patient about the importance of controlling high blood sugar with proper medical management to avoid uncontrolled diabetes-related severe complications like EPN.

## References

[1] Misgar RA, Mubarik I, Wani AI, Bashir MI, Ramzan M, Laway BA (2016). Emphysematous pyelonephritis: a 10-year experience with 26 cases. Indian J Endocrinol Metab.

[2] Montelongo-Rodríguez FA, Robles-Torres JI, García-Saucedo J, Ruíz-Galindo E, Pacheco-Molina C, Gómez-Guerra LS (2021). Emphysematous pyelonephritis and emphysematous cholecystitis: a result of uncontrolled type 2 diabetes. Ochsner J.

[3] Ubee SS, McGlynn L, Fordham M (2011). Emphysematous pyelonephritis. BJU Int.

[4] Abdul-Halim H, Kehinde EO, Abdeen S, Lashin I, Al-Hunayan AA, Al-Awadi KA (2005). Severe emphysematous pyelonephritis in diabetic patients: diagnosis and aspects of surgical management. Urol Int.

[5] Chen KW, Huang JJ, Wu MH, Lin XZ, Chen CY, Ruaan MK (1994). Gas in hepatic veins: a rare and critical presentation of emphysematous pyelonephritis. J Urol.

[6] Derouiche A, Ouni A, Agrebi A, Slama A, Slama MR, Chebil M (2008). Management of emphysematous pyelonephritis based on a series of 21 cases. [Article in French]. Prog Urol.

[7] Baby N, Thoman M, Sunny B, James A, Sivakumar T (2018). Case report on emphysematous pyelonephritis with diabetic ketoacidosis. J Clin Transl Endocrinol Case Rep.

[8] Aboumarzouk OM, Hughes O, Narahari K, Coulthard R, Kynaston H, Chlosta P, Somani B (2014). Emphysematous pyelonephritis: time for a management plan with an evidence-based approach. Arab J Urol.

[9] Morioka H, Yanagisawa N, Suganuma A, Imamura A, Ajisawa A (2013). Bilateral emphysematous pyelonephritis with a splenic abscess. Intern Med.

[10] Momin UZ, Ahmed Z, Nabir S, Ahmed MN, Al Hilli S, Khanna M (2016). Emphysematous prostatitis associated with emphysematous pyelonephritis and cystitis: a case report. J Clin Urol.

[11] Huang JJ, Tseng CC (2000). Emphysematous pyelonephritis: clinicoradiological classification, management, prognosis, and pathogenesis. Arch Intern Med.

[12] Lu YC, Chiang BJ, Pong YH, Huang KH, Hsueh PR, Huang CY, Pu YS (2014). Predictors of failure of conservative treatment among patients with emphysematous pyelonephritis. BMC Infect Dis.

[13] Wang JM, Lim HK, Pang KK (2007). Emphysematous pyelonephritis. Scand J Urol Nephrol.

[14] Punatar C, Jadhav K, Kumar V, Joshi V, Sagade S (2019). Neutrophil: lymphocyte ratio as a predictive factor for success of nephron-sparing procedures in patients with emphysematous pyelonephritis. Perm J.

[15] Wang Q, Sun M, Ma C (2018). Emphysematous pyelonephritis and cystitis in a patient with uremia and anuria: a case report and literature review. Medicine (Baltimore).

[16] Weintraub MD, Winter Iii TC (2021). Emphysematous pyelonephritis in a diabetic patient. BMJ Case Rep.

